# Electro-Physiology

**Published:** 1851-02

**Authors:** James Bryan

**Affiliations:** Professor of Institutes of Medicine and Medical Jurisprudence in the Philadelphia College of Medicine, Member of the American Medical Association, President of the Medico-chirurgical College of Philadelphia, Professor of Surgery in Geneva, N. Y., &c., &c.


### BUFFALO MEDICAL JOURNAL

AND

### MONTHLY REVIEW.

VOL. 6. FEBRUARY, 1851. NO. 9.

### ORIGINAL COMMUNICATIONS.

ART. I.—*Electro-physiology. Report on the Memoirs of M. E. Du Reymond (of Berlin,) on Electro-physiological Phenomena, presented to the Academy, (Commission M. M. Magendie Becquerel, Despretz Rayer, Pouillet Reporter.) Translated from the French of the "Comptes Rendus," (No. 3, July,* 1850.) By JAMES BRYAN, M. D., Professor of Institutes of Medicine and Medical Jurisprudence in the Philadelphia College of Medicine, Member of the American Medical Association, President of the Medico-chirurgical College of Philadelphia, Professor of Surgery in Geneva, N. Y., &c., &c.

Three classes of phenomena are observed in Electro-physiology :

1st.—Those developed in electrical fish:

2d.—Those which are the result of some external known cause, as the commotion due to the shock of the Leyden jar, to the current of the pile, &c. We denominate these, phenomena of external currents:

3d. Those which result from unknown causes, and in which we, however, observe all the electric characters. These are denominated phenomena of organic currents.

This classification has nothing definitive; it is evidently transitory ,and we adopt it to avoid confusion. When the currents which we denominate here organic, shall be better studied, when their causes, now unknown or imperfectly understood, shall have been analysed, or even circumscribed with more care, we may then establish rational distinctions. At the same time, there need be no difficulty in comprehending them under a common denomination, especially, with the explicit reservation, that the denomination of *organic currents* prejudges nothing in reference to the producing cause; it is experience and discussion which must decide whether this cause be organic or in-organic; whether it is interior or exterior, and in the latter case, the corresponding phenomena will be arranged among those of the second order.

We know, for the rest, that Nobili has used the expression of *proper current* of the frog: that Matteucci uses the *muscular current*, and that Du Bois Reymond has employed the terms, *laws of the muscular current*, *laws of the nervous current*, &c.

We will include these expressions under the general one of *organic currents*, as embracing them all: but always under the reserve above expressed.

The memoirs which Du Bois Reymond has presented to the Academy refer only indirectly to electric fishes, we will then pass by these phenomena, in order to attend exclusively to the two other branches of electro-physiology.

1st.—Phenomena of External Currents.

The electric commotion was the first phenomenon which manifested the action of electricity on living bodies. Observers were not long in discovering that this commotion might assume all degrees of energy, that it might be strong enough to kill like powder, or weak enough to produce contractions or sensations almost imperceptible.

The pile of Volta, according to its strength, produced all the effects obtained from bodies simply electrified, or from the most powerful batteries. To reappear in another form, and in different conditions, the phenomenon did not retain all its primitive complexity; only, science may hope that, possessed of new means, she will one day be able to penetrate further than this singular action, and that she will discover finally the modification which the electric forces impress upon the organic forces, to determine the irresistible and instantaneous movement which constitutes the commotion.

We will not here recount the history of the different opinions which have been held on this subject, nor the various experiments which have been performed; but it will be proper to refer, in a few words, to the principal facts which enter into the investigation which we are to make, and see their bearing on the subject.

At the origin of galvanism, and in operating upon the frog, which we will denominate *galvanic*, to indicate that it has been prepared in the manner of Galvani, Volta was among the first who conceived from the contractions, important facts, viz:

1st.—That the contraction is almost certain in *direct current*, that is, that which courses the nerves in the direction of their distribution; and that they seldom take place in *inverse currents*; or in passing from the circumference to the vertebral canal.

2d.—That the contraction observed at the first passage of the current ceases, after a short time, and sometimes that it reappears at the moment that the current has ceased by the division of the circuit.

3d.—That the galvanic frog always becomes insensible to the current, either direct or inverse, when it has continued twenty-five or thirty minutes; but that it remains sensible to the contrary current, and that it may thus recover its sensibility, if in lieu of being subjected to a contrary current, it is allowed to repose a few minutes; from which fact the name of *alterative voltaics* has been given to this phenomenon?

In 1800, M. Le Hot observed that if the *direct current* induces the contraction at the first moment of application, it is the *inverse current*, he thought, induces the contractions at the instant of breaking the circuit; that the same thing occurs in reference to the taste, viz: it is excited on closing the circuit, if the current passes from the tongue to the metal, and at its rupture, if the current passes from the metal to the tongue.

In 1816, M. Bellingeri, in confirming the results in relation to the contractions, added this important circumstance, already intimated by Pfaff, Creve, and some others, viz: that these contractions take place with the same regularity and the same force, when, in place of passing the direct current or inverse currents through a muscle or nerve, we pass then, through a certain extent of nerve which has been isolated; if the direct current be applied, it will be transmitted from the proximal extremity of the nerve to the proximal extremity of the muscle; if the inverse current be applied, the positive wire on the contrary is to be applied to that portion of the nerve nearest to the muscle, and the negative wire to that part nearest the vertebra.

In 1817, M. Marianini, by very ingenious and well directed experiments, was led to announce the following proposition, that the *direct current* induced, at the instant of contact, a contraction, and a sensation at the instant of its cessation, and that the inverse current, produced in inverse order the two phenomena of contraction and sensation; but, to the present time, the experiments of others have not confirmed this law in full. It is difficult to secure all the conditions necessary to produce the same result.

In 1829, Nobili stated that the galvanic frog, on account of great vigor, experienced contractions almost as forcible at the moment of closing the circuit, whether the current was direct or inverse, and that it was only when it was somewhat weakened, that the law observed by Le Hot, was presented with regularity. He has stated another fundamental fact; it is that while acting on *solitary and isolated nerves*, by means of either the direct or inverse currents, with a certain degree of intensity, *tetanic convulsions* or an *electrical tetanus*, analagous, perhaps, to ordinary tetanus, with the simple condition of establishing and breaking the circuit, stroke after stroke, at short intervals; from it follows, says he, "that a continuous current tends to weaken the nerves, and an intercepted one, to excite them."

In 1844, M. M. Longet and Matteucci, in isolating the anterior root of the spinal nerves, in the horse, dog, rabbit and frog, to subject the longest portion possible to the direct and inverse currents, announce that the results obtained were exactly the reverse of those presented by mixed nerves; that is to say, that in the anterior spinal root, the contractions took place, only at the commencement of the inverse current, and at the discontinuance of the direct current.

In 1844, M. Matteucci having concluded, from some interesting experiments, that under the influence of the direct current a nerve loses sooner its sensibility, than under the influence of the inverse current of the same force, recurring later, in 1846, to this first result, and after having applied the mechanical means of experimenting, which we will allude to, he was led to make the following general proposition:

"That the electric current circulating in mixed nerves of a living animal, or recently killed, varies the excitability of the nerve; if the current is direct, the excitability is diminished and destroyed, while this excitability is preserved and augmented by the passage of the inverse current."

In 1847, M. Matteucci, continuing his experiments on the comparative effects of the inverse and of the direct currents, gives as the consequence of these investigations, this result, worthy of attention, viz: that in separating the two members of a galvanic frog, and in so disposing them, that the nerve of one shall be traversed by the direct current, and that of the other by the inverse current, furnished by a pile of Farradis, of 15 or 20 elements, the first becomes insensible, in conformity to the observation of Volta, after twenty-five or thirty minutes; then if we continue passing the current again for a few minutes, to break again the circuit, it happens that at the instant of the rupture of the member which was traversed by the inverse current, instead of being insensible, it enters into a tetanic contraction, which is suspended if the current be again passed in the same direction, but which will persist for several minutes if the division of the circuit be continued.

Such are the series of results which appear the most important in reference to the effect of exterior electricity on organic nature. We do not say that they are incontrovertable, for some of them appear contradictory; but many of them are based upon experiments whose accuracy has been tested by different observers. While others require additional confirmation.

It was necessary to recite these facts, without which we could not explain those which are connected with what we have denominated organic currents, of which we now proceed to speak.

2d.—Phenomena of organic currents.

Every one, at the present day, is familiar with the names of Galvani and Volta. Everybody knows also, of the memorable discussion carried on by these illustrous founders of electro-magnatism, more than half a century ago.

The question was this: are the contractions of the galvanic frog due to its own, or to electricity from without. Galvani advocated the electricity of the frog. Volta, the external electricity; and it is a curious fact, well calculated to inspire those who seek truth, with prudence and modesty, even those who have the greatest penetration and most firm convictions, and these two opinions, apparently exclusive and contradictory, are probably nothing more than a mixture of error and truth. In certain cases, the contraction results from external electricity, as Volta says, but this electricity has not the origin which he assigns to it. In other cases, the contraction results, perhaps, from the electricity of the tissue, according to Galvani, but its source is unknown, and we will see, further on, the opinions which we have gathered in this matter.

As the opinion of Volta prevailed for many years, it is not to be wondered at that before the voltaic pile and its effects, the opinion of Galvani was neglected, that it was almost abandoned to oblivion; its time had not yet come; not only had it not attained confidence and support by any discovery, but it had been kept in a state of impotence disabling it from accomplishing anything.

It followed from this, that the electro-magnetic action was known, that the multiplier was invented, and finally perfected by the principle of compensation.

Then Nobili, the author of this ingenious finish, who gave to the Galvanometer a sensibility analagous to organic sensibility, had scarcely finishe his apparatus when he made a most happy application of it.

He desired to know whether his galvanometer, more delicate than the galvanic frog itself, in its impressions, would indicate the most feeble electric forces. It is in this comparison of his two apparatus, of dissimilar structure, subjected, however, to a common law, affected, each of them by the same influence, that Nobili has demonstrated a new fact, which would revive the theory of Galvani. The memoir of Nobili is dated at Reggio, the 3d of November, 1827; it was soon followed by another of the 1st of November, 1829; these two memoirs must be considered among the most remarkable of those which have appeared on this subject, as well for the new truths which they contain, as for the precision with which they are described. Without making a complete analysis, we will present their general facts as follows:

1st.—The galvanic frog has a peculiar current directed from the muscles to the nerves, or from the feet to the head.

2d.—In placing successively, one after an other in the same order, several galvanic frogs, we obtain a pile, whose tension increases with the number of elements, as is demonstrated by the increasing deviations of the needle of the Galvanometers.

3d.—We determine the presence of feeble external currents and their direction, by causing them to pass through a free portion of the nerve of the frog; the contractions which it induces, shows the current to be direct or inverse, according as they take place at the junction or separation of the current.

We should bear this observation in mind, to understand with what care Nobili has himself indicated the habit of the frog, which M. Matteucci afterwards, denominated the *galvanoscopic frog*, after finishing its reparation, and which Du Bois Reymond has since called the *rheoscopic frog.* We prefer the last name because it is less liable to be confounded with the "*galvanic frog*,' which we have adopted as indicating the frog prepared after the manner of Galvani.

After these researches, of so much interest, due to the rare sagacity of Nobili, those of Matteucci come next in the order of dates, which were conducted at the same time, on the subject we are treating of, and upon electrical fishes; but as already indicated, the latter subject will not now be treated of.

M. Matteucci, during a period of ten years, beginning at 1838, has published a large number of memoirs, in which he exhibits laudable and persevering efforts, to obtain general conclusions on the phenomena of electricity, as manifested in cold and warm blooded animals.

He enjoys the double merit of having instituted, in this respect, a large number of experiments which are consulted with interest, and of having contributed greatly to attract attention to these remarkable phenomena. Among the various conclusions to which he has arrived, in his labors, published in his memoirs in 1841-42-43, and in his treaties on Electric-phenomena published in 1844, there are two which appear to be the most exact of the new truths to which he has been led, viz:

1st.—That in all cold and warm blooded animals, either living or recently killed, there is a muscular electric current directed in the muscle itself, *from its interior to its surface.*

2d.—That a *rheoscopic frog* exhibits contractions when its nerve is placed in contact with the muscle of another frog, or with that of a rabbit, and produces in the muscle decided contractions, either with the aid of an exterior current or of mechanical actions.

It is this last phenomenon which has since received the name of *induced contraction.*

But the first of these propositions removes immediately the question whether the *muscular current* of Matteucci, is anything else than the (current proper) tissue current of Nobili; after reiterated attempts, it appears that Matteucci has not been able to establish any well defined distinction between these two.

In reference to the second proposition, that of the *induced contraction,* it establishes a new and important fact, but it is to be regretted that Matteucci has not recognised the consequence which has been suggested to him on this subject by our friend M. Becquerel, and that Matteucci has taken care to assign it to himself after his memoir. (Annales de chimie et de Physique, 3 serie, tome vi, page 39.) This idea would doubtless have led Matteucci to more profound observations than those which he has made in order to explain *induced contraction.*

We come, at last, to the researches of Du Bois-Reymond, who commenced in the month of January, 1843, (Annales de Poggendorff,) and has continued with success up to the last communication, which he has made to the Academy. We will attempt, also, in the matter before us, to condense the chief propositions to which he has arrived by different series of experiments, which, if we may judge by those made in our presence, bear in the highest degree the stamp of great precision, marking the skillful experimenter, joined to the skillful Physiologist.

We hope to neglect nothing essential, in presenting under the following form, the laws established by du Bois-Reymond.

1.—The nerves after their section, and during their vitality, that is, during the whole time that they are capable of exciting muscular contraction, or of transmitting impressions, give origin to a current which is perceptible by the galvanometer, and which, outside the nerve, is directed from the surface, or from the longitudinal section to the transverse section.

The intensity of this current depends upon the position and the distance of the points through which the nerve is placed in the circle of the galvanometer; it is not when these points are symmetrical on account of the *equator* of the nervous trunk considered as a cylinder; it is maximum, on the contrary, when one of the points of contact is on the equator, and the other in the centre of the cylinder, that is to say, in the centre of transverse sections.

2.—The muscles of all animals, while they are susceptible of contraction under any influences, manifest a current analogous to that of nerves subjected to the same laws as well in the direction as the intensity.

Of which it should be remarked that certain muscles, such for example as the gastrocnemii and the triceps of the frog, present *natural transverse sections* where the muscular fibre is joined to the tendon, the muscular aponeuroses being nothing more than coverings of the natural transverse sections.

3.—In comparing these different muscles among themselves, we observe that the intensity of the current is in proportion to the amount of mechanical force exercised by the muscle, whether voluntary or involuntary; thus the fibres of the heart, which are involuntary, manifest an energetic current like the muscles of relation, which are all voluntary: at the same time the muscles of the intestines, having but feeble force, manifest a very feeble current.

4.—When a current produced in a gastrcenemius muscle of a frog is observed, and that from any external cause, electric or non-electric, we observe in the muscle repeated muscular contractions, and see in an instant the intensity of the ordinary and natural current to which it gave origin, exhibits a very remarkable diminution of intensity.

It follows that muscular contraction, whatever may be the cause, does not take place, until some considerable change in the interior electric circuation has taken place.

The rheoscopic frog, placed in contact by its nerve, and under the requisite conditions, with this muscle tetanina, exhibits itself corresponding contractions, which result from this diminution of intensity; it is observed itated convulsively if the muscle with which its nerve is placed in contact is itself in a state of convulsion; and if, on the contrary, the contractions of this muscle are distant and successive, the rheoscopic frog in some degree counts them, and measures them by distant and successive movements which correspond. This fundamental fact presents a direct explanation of the *induced* contraction of Matteucci.

The rheoscopic frog, which has only its nerve placed in the circuit, presents the same phenomena.

5.—When we observe on the galvanometer a current produced by the nervous trunk, which enters, for example, the circuit, only with half of its length, touching on one side by its transverse section, and on the other by the points of its equator, and we attempt to produce different actions on the end of the free half which is without the circuit, we see that the ordinary and natural current to which it has given birth, exhibits a diminution of intensity, analogous to that which is seen in the muscle at the moment of contraction. The actions which we induce on the free extremity of a nervous trunk, may be either a direct or the inverse current, either a cauterization, an intoxication, or a mechanical bruising.

It follows that the local actions which they transmit, either to the muscle or to the nervous centre, if the nerves be not detached the one from the other, appear effectual in modifying the electric condition of the nerve in portions even where it is not directly affected.

6.—After having cut, near the pelvis, one of the sciatic nerves of a whole and living frog, it may be so disposed, that by each of its inferior extremities it enters into the circuit of the galvanometer, and closes it, and no electric phenomenon appears. Inducing the absorption of nitrate of Strychnia, tetanus is manifested, and is manifested only in the inferior extremity where the nerve has not been cut; at the instant that the needle of the galvanometer has a current which is without, directed from the contracted member to that which is not, and which is consequently a direct in the contracted member.

Such is the view which we can now present of the principal results of du Bois-Reymond.

Each of these propositions is nothing more, as already stated, than a general enunciation of a great number of comparative experiments, performed and arranged with care. M. du Bois-Raymond has repeated before us all those experiments which we consider the most important, and he introduced, at our request, all the modifications which we thought of.

It will be readily understood how much zeal and penetration would be necessary to appreciate the profound reasoning, in a subject comparatively new, where it may be necessary, in some sort, to create means of observation, modes of experiments, as well as experiments themselves.

M. du Bois-Reymond had, as a point of departure, the results of Nobile and those of Matteucci, previous to 1843, which we have given above. From these one may judge of the progress which he has made in this rising portion of electro-physiology, and at the same time the important direction he has given to experimental science.

Thus far we have spoken of the works of M. du Bois-Reymond exclusively, in reference to experiments on animals, and the laws which he has deduced from them; we must now speak of another observation which merits particular attention.

Every one knows that we design to speak of the current which appears to be developed in the human body, endowed with full life, at the moment of the contraction of the muscles of the arm by voluntary action.

This new fact, discovered by du Bois-Raymond, is not less positive, nor less clearly confirmed than the preceding; we add, besides, that it is not the less general; that is to say, the first person who comes, when we explain to him the mode of procedure, will without fail produce a greater or lesser deviation in the needle of the galvanometer; at the same time, the intensity of the effects seems to be due rather to the power of the will, than to the force of the muscular contraction.

We will present this fact in a distinct point of view for two reasons; because it raises an important question, and because it has been the principal object of the communications which M. du Bois-Reymond has made to the Academy. In his first note we read these words: "The object of this note is to inform the Academy of the series of experiments which have terminated by leading me to the discovery of the development of an electric current in the muscles of a living man at the moment of contraction."

"The great question which this fact solves, then, is this: in the living man, is there an electric current developed at the moment of contraction?"

We proceed to state that the production of a current is incontrovertible; that this current is demonstrated by the galvanometer with less proof than those developed, under the requisite conditions, when the muscles or the nerves are introduced to the circuit. It is already a fundamental point, but this is not all; it remains to see whether this current, of which the galvanometer proves the presence, is, in effect, developed in the muscles, and if it be the necessary result of their contraction.

Now there is here abundant material for controversy; we do not expect to decide the question; we will attempt only to discuss it, and to indicate the doubtful points. To simplify this examination, to proceed by degrees from the more simple to the more complex phenomena, we must commence by taking a bird's eye view of the organic currents in general, in order to separate, as far as we can, that which is known from that which is unknown in their origin and cause.

We have stated that the tissue current (courant propre) of the frog was advocated by Galvani and opposed by Volta. They agreed in the fact, each admitted the simple contraction of the frog, that is to say, that which was produced by the contact of a nerve, and by certain points of a muscle; but, while Galvani attributed this phenomenon to the tissue current, Volta explained it by the heterogeniety of the elements placed in contact, and by the *electro-motive force*, which in his theory, is its result.

The progress of science, especially since the discovery of electro-magnetism, has by degrees brought to light a new force, suspected before by Fabroni and other experimenters, which is capable also of developing electricity and of generating currents; this force is *chemical action*. As soon as this power was well established, it immediately invaded the vast domain which the genius of Volta had attributed to the electro-motive force, and of which, step by step, it has taken real and sovereign possession.

The electro-motive force, such at least as is now admitted, has disappeared almost entirely, and with it have disappeared also the explanations given by Volta of the simple contraction of Galvani.

But in examining in its turn chemical action itself, do we arrive at this conclusion: Is this the sole cause capable of producing these electric currents? This question has long since been answered in the negative.

In admitting the existence of ordinary electricity developed by friction and pressure, there are two great classes of phenomena which belong evidently to chemical action, viz: the electric phenomena which are presented by crystals analogous to tourmaline, and by the thermo-electric phenomena.

Has science, arrived at that point, or not, at which it can enumerate, without cavil, all the several causes which give rise to the development of electricity? Can it say that every electric current is induced essentially by the causes at present known and established? We do not think so. On what ground indeed is this to be the case? The actual causes are diverse and without explanations; we know that they are effectual, but we do not know why they are effectual; we presume that there is among them a certain connection, we neither know in what this consists, nor in what it can consist; we are struck with their diversity, but are ignorant of the principle which doubtless binds them together. Now, in this ignorance, almost absolute, how can we affirm that this primitive principle shall never manifest itself under new forms and aspects, which are at present hid from our view?

Our efforts should then have a double object; to distinguish analogous phenomena and attempt to search out their laws, and mark, as well as we can, the character of the cause which produces them: to seek new phenomena to discover new causes, or to investigate more thoroughly the causes already known.

The phenomenon above stated, so well observed by Nobili, although not new in every respect, is not the less, under the present aspect, a discovery of much importance.

In the first place because it teaches, for the first time, this fundamental fact, that the galvanic frog presents a current capable of causing a deviation in the charged needle, a regular current, in a definite direction, and dependent on the organic sensibility, in this sense at least, that it follows or marks all the phases; increasing when it increases, and ceasing when it ceases.

Secondly, because the cause of such a current, instead of disappearing with evidence of the mode of experimenting, appears to be hid in the depths of organic nature itself.

Indeed, Nobili has indicated in passing, that the tissue current (courant propre) may perhaps have a thermo-electric origin, but he has not demonstrated it, and it should be added, no experimenter has attempted to demonstrate it, so slightly are the analogies in favor of this opinion.

After Nobili, it has been thought that the tissue current owes its origin to chemical actions; but as yet this opinion does not appear to have received sufficient support. Chemical action has its infallible criterion; when invoked we are bound to show on what it is exercised and what it accomplishes. Now, in the matter before us, no one has pointed out either the elements in action or the products formed. There is indeed a necessary distinction on this point; if the tissue current is the result of a chemical action, is it not important to know whether it is an internal or external action? That is, whether it is a simple reaction of the constituent organic elements, without the influence or concurrence of any ponderable foreign agent, or whether it is an action exercised on the organic body by exterior means which are in contact with it.

In the latter case, the galvanic frog, or the organized body in general, is simply analogous to plates of zinc and copper, which do not possess in themselves the power of developing electricity, and which demand, to obtain this power, to be placed in contact with an acid or other conducting medium capable of combining with them.

In the first case, on the contrary, the organic body possesses in itself the power to generate currents; it possesses it either from its nature, or from its structure, or from the chemical reaction of its proper and constituent elements.

We are thus forced back to the discussion between Galvani and Volta; the ground is not the same, the arguments and proofs have changed character, but at bottom it is the same thought.

It should be remarked, again, that this distinction applies not only to the phenomena before us, but also to electrical fishes themselves, that is to say, in general to all the electro-physiological phenomena which are not the evident result of electricity whose source is without, and which is made to pass artificially into organic bodies.

Electro-physiology scarcely exists, it is connected with phenomena infinitely amplex, which appear to be one of the links which bind inorganic with organized nature, it must in common with the other experimental sciences appeared, with methodic and reflecting care, the ground on which it proposes to be built.

For this reason we insist upon this primary and old question: the currents which are manifested in the tissues of life, or of recent life, have they an internal or external cause, a known or an unknown cause? It is to be regretted that this question has not been approached in a manner very explicit by the above named experimentors; they would, doubtless, have conceived experiments directly to the purpose, and calculated to remove all doubts.

The following, then, may be drawn from their experiments, although they were not instituted for that purpose:

M. Matteucci, pursuing the idea of Nobili, and perfecting it, has constructed a pile by the simple juxtaposition of organic elements, without any intermedium, the two extreme elements alone, being in connection by means of fluid conductors; now the deviations by these piles appear to indicate that if the currents were due to an external chemical action, it should follow, that when the organic elements were in contact, the chemical action to explain why it was developed in the second and not in the first place.

Many other experiments must be resorted to with the same view.

In the actual condition of things, the committee have not been unanimous in forming a definitive conclusion; it is disposed to say only, that the whole of the phenomena present a great probability that the organic currents are not the effect of an external chemical action, but that it will be well to present proofs more positive than those which have as yet been presented.

In supposing this first question resolved in the sense that it ought to be, another presents itself which is not eventful to every body, and which has already been the subject of many discussions. It is this: the currents developed, do they originate in an internal chemical action, or from the nature only of the structure of the tissue submitted to their particular forces?

Nothing is more evident than the prodigious variety of the chemical phenomena which are brought to view in an organized being at each instant of its existence. Among these, there are some which cease immediately with life, and others which continue under the influence of forces with which we are but imperfectly acquainted; these, considered as simple chemical phenomena, do they present a clear exposition? either the cause should be the same as when they touch the fluid conductor, which is different in its nature.*

M. Du Bois-Reymond, having demonstrated that the nerves give birth also to currents, whose laws he has established, it should follow, that the action of the fluid conductor, with which he had placed them in contact, should be the same on the substance of the nerves as on the substance of the muscles; and as besides, we can make piles with nerves as well as muscles; it should follow the more, that the transverse and longitudinal sections of the nerves should excite, among themselves, a chemical action, such as they exercise on the fluid conductor.

We regret not having demanded of Du Bois-Reymond the performance, before us, of the following experiments, which he has probably had occasion to perform in the course of his researches: a nerve on which a ligature has been applied, retains a sufficient amount of conductability; it may be introduced into the circuit in two modes, either by the connection of the ligature itself, or by leaving this out, and placing the nerve in such a position that it shall touch on one side by its transverse section, and on the other by its longitudinal section. Now, should it happen that in the first case, it presents nothing, and that in the second case it exhibits an ordinary current, it would be difficult to attribute the current to an external chemical action, for it would be very difficult, of the contractability which exists in the muscles, of the sensibility in the nerves, or of other propor-

* There is an obvious obscurity in this passage which we are unable to clear up. The manuscript of the translator is literally followed, but something is evidently omitted.—Editor.

tions which still remain, for a longer or shorter time, according to the rank which the animal occupies in the scale of being.*

It appears to us, that, in answering, in the affirmative, in accepting this explanation as sufficient, we cause chemical action, which is so clear and precise, to descend to the rank of occult and intangible causes, we cause it to loose its essential character which it has obtained from positive analysis and its effects.

But if we cannot now hold that the chemical actions, which succeed life, explain all the persistent organic properties, can we say that they explain all the electric phenomena which are observed on the galvanometer; and which diminish with the remains of vitality. On this point, opinions are divided; no person, doubtless, holds that the chemical actions, which are developed, are not accompanied with a disengagement of electricity; but some, contented with this general idea, regard as very probable, if not as certain, that this electricity is the cause of the organic currents; others are doubtful, and wait for further analysis and study of the chemical actions, and while waiting, doubt, more or less, whether, by this means, a complete and explicit explanation of the intensity and all the other characteristics of the organic currents, will be obtained.

This is, then, but one question to be solved, a general question, in which we must not loose sight of the fact, that not only have we to seek the cause of the currents observed in the nerves and muscles, but that there are two phenomena besides to be explained, viz: the intermittent diminutive force which the muscular current undergoes during the contraction of the muscle, and the modification observed in the nervous current during the excitation of the latter.

We must, moreover, make another remark: the laws given by Du Bois-Reymond, are presented, in general, either by layers of muscles and nerves connected with a painful existence, or by detached portions whose separation has been so recent, that they still retain something of what we denominate *organic sensibility*. But are the incisions of the scalpel without influence? Does not the mutilation itself enter into the phenomena observed? The exudation of fluids, altered currents by endosmosis or otherwise, do they not take some part? These questions demand serious examination; they should be discussed and solved, concluding from a part to the whole.

Here, induction alone is powerless, we want proofs—proofs positive—to

---

* See note on the preceeding page.

be authorized to explain the entire muscular system, and especially the whole nervous system, that which results from a separate portion of nerve and that which is the result of the scissors.

Thus, then, in fine, our opinion of the cause of the organic currents in general is as follows: their cause is unknown.

1st.—It is probable that these currents result from exterior chemical action.

2d.—It has not been demonstrated that they result from an interior chemical action; there is here a question for solution, and as it shall receive a positive or negative answer, the ulterior consequences vary.

Let us return to the current which appears to follow or result from the contraction of the arm.

These are the elements of the discussion,

1st.—If, according to the experiments mentioned in the sixteenth proposition, (of facts observed by Du Bois-Reymond,) a frog does not present a sensible current, it is that the inferior extremities tend to produce equal and opposite currents.

2d.—When, having cut one of the sciatic nerves to paralyze the limb, tetanic contractions are produced in the other, the intensity of its current diminishes in conformity with the fourteenth proposition; then the current of the limb paralyzed or condemned to respose, becomes predominant, and the galvanometer renders it visible with the direction it should take; that is to say, that it is *direct* in the tetanised member.

3d.—When both arms are placed in the galvanometer, none but accidental effects are observed, dependent, doubtless, on the cutaneous condition of the fingers, which touch the conductors; when these irregular effects have ceased, and the needle of the galvanometer has become stationary, voluntary contraction of one arm is induced; when the needle of the galvanometer is at once effected, its deviation always shows an *inverse* current; that is to say, directed, in the contracted arm, from the hand towards the shoulder.

Now, if we examine these last propositions, to see their connection, a difficulty will be found, thus; between the second and third, there is a certain analogy; there, then, is a leg which contracts artificially, here an arm which contracts voluntarily; but why is the current *direct* in the first case, and *inverse* in the second? This is an important point; it is to be regretted that Du Bois-Reymond, who has himself taken care to point out this difference, this constant inversion in the course of the current, has not felt the necessity of explaining the reason; while this explanation remains unmade, we must doubt whether there is a necessary, or even any, connection between the third and the second experiments. According to the principles of Du Bois-Reymond, the effect of a sustained contraction, is not to induce a current, but to enfeeble and to suspend, by intermission, a pre-existing current; there must, then, be a pre-existing current, as indeed there must be, two equal and opposed, which neutralize each other, while the needle of the galvanometer is at Zero; the one must be essentially in the arm, which contracts, and it is this which the contraction enfeebles; the other, for the same reason, must be in the other arm, and this one, the contraction renders dominant. Thus the current, observed at the instant of contraction, is not developed in the arm contracted; it is, on the contrary, pre-existant in the quiet arm, and it exhibits itself only because it ceases to be entirely neutralized.

If the question should be put in these terms, it appears to us, to assimilate this experiment to the preceding; one condition must be fulfilled, that is, to demonstrate clearly that the muscles of the arm of a man, in a state of contraction, if considered as in their natural condition, are disposed in a way to induce a direct continued current, going from the shoulder to the hand, and that they induce this current according to the laws of longitudinal and transverse sections. This condition is indispensable, while if it is not answered, the experiments cannot be assimilated; we cannot, and ought not to regard the third proposition as being a consequence of the first. But let us admit, for on instant, this first difficulty to be removed, that the form of the muscles of the arm which are here engaged, that their structure, their interlacement, their absolute and relative condition, conduce to the desired conclusion, that is to say, that it suffices to apply to it the muscular current, to demonstrate that in composing the directions and intensities, we obtain as a final result, a continued current from the shoulder to the hand: will the whole question be resolved? Should we consider as certain that the third experiment is identical with the second, and that it is explained fairly by the same cause? We do not think so. There will still be doubts depending on the diversity of the conditions and of the complexity of the problem; but the time has not arrived to resort to them and to discuss their value.

In fine, the current which appears to pertain to the muscular contraction of a living man, is a very curious phenomenon. While applauding this discovery of M. Du Bois-Reymond, while, according to it, perhaps, intimate connections with other electro-physiological phenomena, of which he has so skilfully studied the laws, we cannot admit that these connections have as yet been demonstrated in a conclusive manner.

We will not conclude this Report without making a reflection designed to encourage M. Du Bois-Reymond, to stick closer and closer to those vigorous methods which have couducted him to so many new facts. It is impossible not to remark some words which he uses at the commencement of his first Memoir, and in which he announces " that his researches border on a positive theory of the nervous agent, and of the motive power of the muscles."

While the text of his communications does not approximate the discusson of any theory, it contains facts whose correctness and importance we have attempted to point out.

The theory announced in the words quoted, we have not examined; we can only consider them as a special point of view, an abstract idea, a future thought to be placed before the Academy. It appears quite natural that Du Bois-Reymond should expect for himself great things, from the fecundity of the new career in which he has entered to investigate truth, the first approaches of which he has explored with a success worthy of praise. Thanks to the modes of observation he has conceived, he has discovered facts of a certain character, which will add much to science; but nothing demonstrates, as yet, that they are other, that is to say, truths which explain the *nervous agent and the motive power of the muscles.* If, one day in future, after long and laborious reseaches, these truths having been subjected to the crucible experience, remain, science may then be said to have made a bold stride onwards.

Finally, your committee have witnessed with the liveliest interest the experiments of Du Bois-Reymond. They bear testimony to the great precision of those performed before them, and add, that they expect much from researches still more exact, which will be made in this direction.

In conclusion, we propose to the Academy to thank M. Du Bois-Reymond, and to congratulate him on his diverse series of facts which he has demonstrated by experiment.

The conclusions of the report were adopted.

